# Literature Review and Case Histories of *Histoplasma capsulatum* var. *duboisii* Infections in HIV-infected Patients

**DOI:** 10.3201/eid1311.070665

**Published:** 2007-11

**Authors:** Pierre Loulergue, Frédéric Bastides, Véronique Baudouin, Jacques Chandenier, Patricia Mariani-Kurkdjian, Bertrand Dupont, Jean-Paul Viard, Françoise Dromer, Olivier Lortholary

**Affiliations:** * University Paris V, Paris, France; † Hôpital Necker-Enfants Malades, Paris, France; ‡ Centre Hospitalier Universitaire Bretonneau, Tours, France; § Hôpital Robert Debré, Paris, France; ¶ Institut Pasteur, Paris, France

**Keywords:** HIV infection, African histoplasmosis, emerging disease, antifungals, synopsis

## Abstract

African histoplasmosis caused by *Histoplasma capsulatum* var. *duboisii* is an invasive fungal infection endemic in central and west Africa. Most of its ecology and pathogenesis remain unknown. *H. capsulatum* var*. capsulatum* is an AIDS-defining opportunistic infection in HIV-infected patients who are living in or have traveled to histoplasmosis-endemic areas. In contrast, reports concerning African histoplasmosis during HIV infection are rare, although both pathogens coexist in those regions. We report 3 cases of imported African histoplasmosis diagnosed in France in HIV-infected patients and a literature review on similar cases.

## Abstract 2

African histoplasmosis during HIV infection is rare.

Human histoplasmosis is caused by 2 varieties of *Histoplasma*. The most common variety worldwide is *H. capsulatum* var. *capsulatum*, which has been reported from many disease-endemic areas where HIV infection is prevalent. Histoplasmosis is more frequent in the United States (Ohio and Mississippi River valleys), but it is not unusual in other parts of the world, such as Africa (*1*,*2*). In the western and central regions of sub-Saharian Africa, *H. capsulatum* var. *capulatum* coexists with another variety, *H. capsulatum* var. *duboisii*, whose ecology and pathogenesis remain almost unknown. Cases due to *H. capsulatum* var. *duboisii* are scarce in Europe, and all are imported (*3*).

Before the era of highly-active antiretroviral therapy (HAART), the prevalence of *H. capsulatum* var. *capsulatum* infections reached up to 30% of HIV-infected patients in hyperendemic areas of the southeastern part of the United States (*4*). The infection occurs more often in patients with a CD4 count <50/mm^3^ and is usually disseminated. For unknown reasons, although HIV infection and *H. capsulatum* var. *duboisii* coexist in Africa, this coinfection remains rare (*5*). We report 3 imported cases of the potentially emerging histoplasmosis due to *H. capsulatum* var. *duboisii* diagnosed in France during the course of HIV infection and a literature review on similar cases.

## Case 1

A 37-year-old man from the Democratic Republic of Congo, who had lived in France since 1980, was infected by HIV-1 since 1987. He was admitted to the hospital in 1992 because of a fever of unknown origin. His physical examination showed a left axillary tumefaction 2 inches in diameter. This mass had already been explored 5 months before. At that time, histopathologic examination disclosed a necrotizing lymphadenitis with epithelioid cells but without caseum. No microorganism was seen after Ziehl, periodic acid–Schiff, and Grocott stainings, but culture was not performed. When the patient was hospitalized in 1992, laboratory tests showed an erythrocyte sedimentation rate of 104 mm, fibrin 4.5 g/L, C-reactive protein 74 mg/L, and a CD4 count of 100/mm^3^ (9%). The adenopathy was surgically removed. Histopathologic examination showed necrosis and large yeasts, and culture grew *Histoplasma* sp. on day 12. Anti-*Histoplasma* antibody detection was negative. No other lymph node, bone, skin, or bone marrow involvement was found.

Itraconazole treatment was started (400 mg/d), but it was switched to amphotericin B (1 mg/kg/d) after 3 weeks because local symptoms persisted. The total dose of amphotericin B was 1,200 mg. Itraconazole (400 mg/d) was then restarted for 1 year. The clinical course was satisfactory, so itraconazole was lowered to 200 mg/d for 3 years. The patient died in 1995 of HIV-related encephalitis despite antiretroviral therapy, including nucleoside reverse transcriptase inhibitors, without recurrence of histoplasmosis.

## Case 2

A 41-year-old man from the Democratic Republic of Congo, who had lived in France since 1981, was infected by HIV-1 in 1994. He received AZT (3′-azido-3′-deoxythymidine) and diethyldithiocarbamate (ddC) when *Pneumocystis* *jiroveci* pneumonia was diagnosed in December 1995. Despite the introduction of HAART in 1996 (AZT, lamivudine [3TC], and ritonavir), his CD4 count remained <50/mm^3^. In mid-1996, nodular cutaneous lesions, a right cervical adenopathy, and a right Bell’s palsy developed. Direct examination of the lymph node showed numerous yeasts with a typical lemon shape and a narrow budding, suggestive of *H. capsulatum var. duboisii*. The culture grew *Histoplasma* sp.

The patient did not respond to itraconazole (400 mg/d). After 1 month, he was given conventional amphotericin B (1 mg/kg/d); severe renal insufficiency developed within 8 days. Treatment was switched to liposomal amphotericin B (3 mg/kg/d) with a dramatic improvement of the symptoms and partial regression of the renal insufficiency. Immune reconstitution inflammatory syndrome also developed; its characteristics were reported previously (*6*). After 1 month, treatment was switched to itraconazole, 400 mg/d, for long-term therapy. Because of a persistent low CD4 count despite undetectable viral load, the patient benefited from several courses of interleukin-2 (IL-2) therapy, which allowed a marked and sustained increase of the CD4 count. Itraconazole was stopped in 1996. His condition remains stable 11 years later, and no recurrence of histoplasmosis has been observed.

## Case 3

A 2-year-old girl from the Democratic Republic of Congo was referred to the hospital in June 2001 for a fever of unknown origin. (She arrived in France in 2001 at the age of 18 months.) Investigations showed *Escherichia coli* pyelonephritis. HIV-1 serologic test results were positive, and her CD4 count was 45/mm^3^. HAART was started quickly, combining AZT, 3TC, and nelfinavir. This treatment resulted in a decrease in, but not elimination of, the viral load and a CD4 count <200/mm^3^ despite appropriate nelfinavir serum concentrations.

In August 2001, a frontal swelling appeared, associated with fever and generalized weakness. Direct examination of a skin biopsy specimen showed large, lemon-shaped yeasts suggestive of *H. capsulatum* var. *duboisii.* Culture of this specimen grew *Histoplasma* sp. Diffuse bone involvement (several lytic lesions of the right humerus, left ulna, both tibias, right fibula) was found on radiographs. Culture of the buffy coat was concomitantly positive for *Fusarium verticillioides.* No involvement of the lungs or lymph nodes was found.

Treatment with liposomal amphotericin B was started but switched to itraconazole after 1 month. Fever relapsed shortly thereafter, as well as the facial tumefaction. Radiographic examination showed several lesions of the skull, and a bone biopsy demonstrated large yeasts on direct examination. Amphotericin B was restarted for 4 months. The patient’s status improved dramatically, and the treatment was switched to fluconazole until September 2003. She did not experience any relapse, and antifungal prophylaxis was discontinued because of the improvement of her immunologic status (CD4 count >200/mm^3^ and undetectable viral load). In July 2007, she is doing well with a CD4 count of 700/mm^3^ and a still-undetectable viral load.

## Discussion

*H. capsulatum* var. *duboisii* is also known as African histoplasmosis because it has only been described on that continent, mostly in central and western Africa. The prevalence of histoplasmosis due to variety *duboisii* has not been established in countries in these regions in HIV-negative patients. Fewer than 300 cases are reported in the literature (*7*). The reason it remains rare, despite the major HIV pandemic in Africa, is unknown. Potential explanations are that patients die from other causes before histoplasmosis develops (*8*) or that variety *capsulatum* is more virulent than variety *duboisii*. This situation is reminiscent of *Cryptococcus gattii* and *C. neoformans*. *C. gattii* is rarely identified in HIV-infected patients, in contrast with *C. neoformans,* whereas both are present in the environment in countries where the prevalence of HIV infection is high (*9*). However, variety *capsulatum* is frequent in Africa. No data on the relative frequency of those 2 varieties has been published. Skin reaction to histoplasmin in histoplasmosis-endemic areas showed a 3% prevalence (*10*), but variety *capsulatum* and variety *duboisii* were not able to be differentiated. Higher prevalence (≈35%) was found in rural populations, especially among farmers, traders, and cave guides (*11*). Histoplasmosis due to variety *duboisii* may be misdiagnosed in those areas because of physicians’ lack of awareness.

The pathogenesis of African histoplasmosis remains unclear. The main route of acquisition could be airborne contamination from the soil, rarely direct inoculation. Variety *duboisii* is classically associated with cutaneous lesions (nodules, ulcers) and osteolytic bone lesions, especially affecting the skull, ribs, and vertebrae (Table 1) (*12**,**13*). Histopathologic examination shows granuloma with necrosis and suppuration. Disseminated disease is not uncommon and can involve every organ; however, the heart and central nervous system are unusual locations. A total of 17 cases have been reported thus far among HIV-infected patients, including the 3 cases described here (*14*–*19*). An additional case has been reported, but without detailed description, in a Ugandan patient diagnosed in Japan (*20*). Among the well-described cases (Table 1), most involved patients with poor immunologic status (mean CD4 count 55/mm^3^), which also occurs with histoplasmosis due to variety *capsulatum* (*21*). Most patients had disseminated infections, and only 4 patients died. The prognosis of disseminated infection in this context is close to the 20% mortality rate reported for disseminated histoplasmosis due to variety *capsulatum* among AIDS patients (*21*), but the few number of cases does not allow us to extrapolate the mortality rate related to variety *duboisii.* Epidemiologic information, clinical manifestations, and outcomes of immunocompetent versus HIV-infected patients infected with variety *duboisii* are compared in Table 2 (*13*). These data confirm the tropism of variety *duboisii* for lymph nodes, skin, and bones. It is noteworthy that the disease is often located in the lungs in HIV-negative patients, whereas HIV-infected patients have substantially more disseminated disease. The latter finding may be explained by immunodepression, poor access to the healthcare system for HIV-infected persons in Africa, and late diagnoses of histoplasmosis.

**Table 1:** Description of HIV-infected patients with histoplasmosis due to *Histoplasma capsulatum* var. duboisii*

Case no.†	Age, y	Sex	Country	Clinical findings	CD4 count/mm^3^	Pathology	Positive fungal culture	Treatment	Outcome
1	20	F	Congo	Skin lesions	NR	Skin	–	AmB 1 mg/kg/d, Itr 300 mg/d	Relapse
2	44	M	Congo	Skin lesions, weight loss, lymph nodes, peritonitis	NR	Skin, pus	–	Ketoconazole 600 mg/d, AmB, Itr 300 mg/d	Relapse
3	41	M	Congo	Skin lesions, weight loss, lymph nodes, hepatomegaly, splenomegaly	NR	Skin	–	AmB	Death
4	65	M	DRC	Fever, weight loss, anemia	NR	Bone marrow	Bone marrow, blood	AmB	Death
5	28	M	DRC	Fever, skin lesions, lymph nodes, weight loss, bone lesions	NR	Skin	Skin	Ketoconazole 600 mg/d	NR
6	31	F	Cameroon	Septic shock	2	Bone marrow	Bone marrow, blood	ABLC 5 mg/kg/d, Itr 400 mg/d	No relapse
7	29	M	Liberia	Skin lesions	NR	Skin	Skin	Itr 200 mg/d	NR
8	43	F	Guinea-Bissau	Fever, weight loss, anemia, abdominal pain	68	Colon	–	Itr 400 mg/d	No relapse
9	30	M	Nigeria	Fever, skin lesions, lymph nodes, anemia	2	Skin	Skin	AmB 1 mg/kg/d, Itr 400 mg/d	Relapse
10	38	M	DRC	Fever, weight loss, lymph nodes	160	Lymph nodes	Bone marrow, lymph nodes	AmB	No relapse
11	26	M	Congo	Fever, skin lesions, lymph nodes	NR	Lymph nodes	–	AmB 1 mg/kg/48 h	No relapse
12	30	M	Côte d’Ivoire	Fever, weight loss, lymph nodes	6	Bone marrow	–	Itr 400 mg/d	No relapse
13	50	F	Nigeria	Skin lesions, bone lesions	NR	Skin, bone	–	Fluconazole 100 mg/d	No relapse
14	45	M	Ghana	Fever, weight loss, splenomegaly	24	Blood	–	AmB 0.7 mg/kg/d	Death
15	37	M	DRC	Fever, lymph nodes	100	Lymph nodes	–	Itr 400 mg/d	Death
16	41	M	DRC	Lymph nodes, skin lesions	50	Lymph nodes	Lymph nodes	Liposomal AmB, Itr 400 mg/d	No relapse
17	2	F	DRC	Fever, skin lesions, bone lesions, blood	45	Skin, bone, blood	Skin	Liposomal AmB, fluconazole	No relapse

*NR, not reported; AmB, amphotericin B deoxycholate; Itr, itraconazole; DRC, Democratic Republic of Congo; ABLC, amphotericin B lipid complex. †Cases 1–14 are from the literature review; cases 15–17 are personal cases; see text.

**Table 2:** Comparison of clinical and microbiologic findings of HIV-infected and immunocompetent patients with histoplasmosis due to variety duboisii*†

Characteristic	HIV positive (n = 17)	HIV negative (n = 20)
Age, y (range)	35 (2–65)	34 (8–62)
Sex (M:F)	12:5	19:1
Visceral localizations		
Lymph nodes	53	65
Skin	59	40
Bones	18	25
Lungs	0†	35†
Gastrointestinal	12	5
Disseminated	85†	55†
Clinical manifestations		
Fever	58†	15†
Weight loss, asthenia, anorexia	54	30
Respiratory symptoms	0	20
Hepatosplenomegaly	12	15
Diagnosis sites		
Lymph nodes	24	45
Skin	48	35
Bone marrow	18	0
Bone	12	5
Gastrointestinal	6	5
Pus	6	25
Lung	0†	25†
Mycologic diagnosis		
Direct examination	100	40
Culture	64	65
Blood culture	12	0
Treatment		
Amphotericin B	66	80
Ketoconazole	12	35
Itraconazole	64	20
Fluconazole	12	0
Outcome		
Relapse	12	40
Death	24	5

*Except where indicated, all values are percentages. HIV-negative patients are from Dupont et al. (*13*). †p<0.05.

Despite its rarity, African histoplasmosis should be kept in mind as a diagnosis in Africa-born patients or travelers to sub-Saharan West and central Africa who have compatible signs or symptoms, even if they are HIV-infected, because the saprophytic phase of this dimorphic fungus should be manipulated in a Biosafety Level 3 cabinet. The laboratory diagnosis is performed by direct examination and culture. Cultures of tissue samples or body fluids are made onto Sabouraud dextrose agar, incubated at 25°C; incubation could be prolonged for up to 6 weeks. The success rate depends on the extent of infection, the source of the sample, and the prompt processing of the sample.

**Figure 1:**
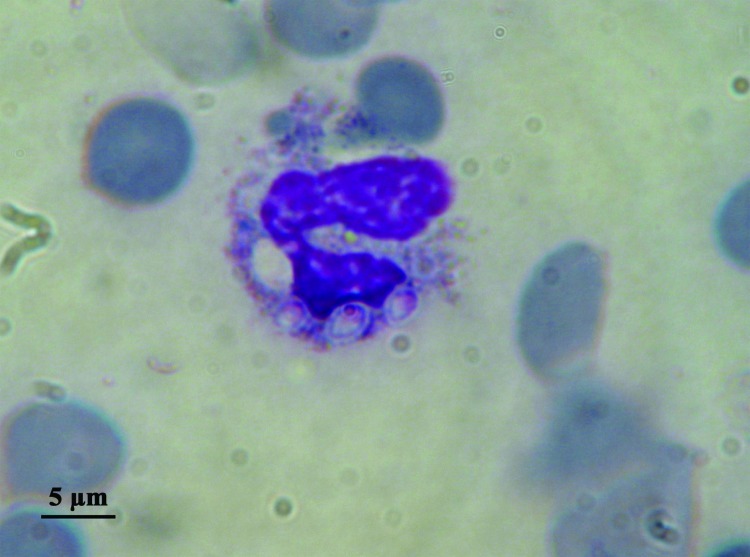
Direct examination of bone marrow smear. Intracytoplasmic *Histoplasma capsulatum* var. *capsulatum.*

**Figure 2:**
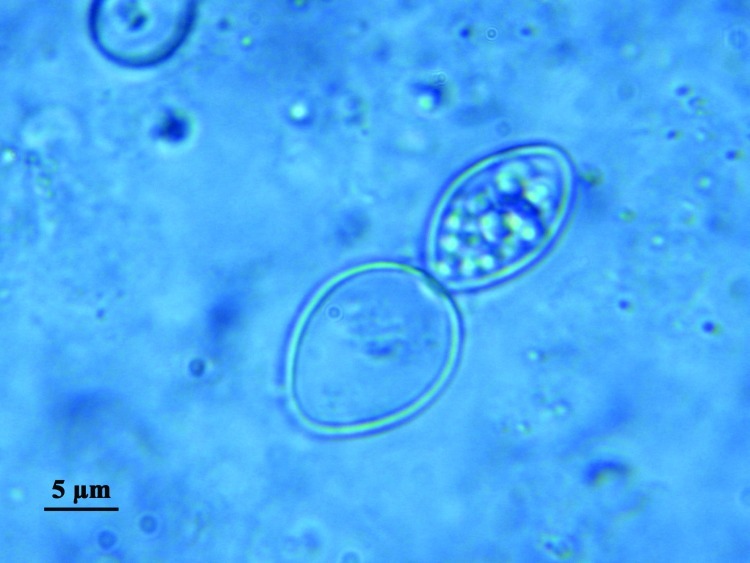
Direct examination of sputum fluid showing *Histoplasma capsulatum* var. *duboisii.*

In addition to differences in clinical manifestations and epidemiology, the 2 varieties can be easily distinguished on observation of the yeast phases present in infected fresh or fixed tissues, whereas the saprophytic phase is identical. Variety *capsulatum* presents as small (3-μm) oval yeasts free or inside histocytes or macrophages (Figure 1), whereas yeasts of variety *duboisii* are large (7–15 μm), globose to ovoid, thick-walled, and typically lemon-shaped with a narrow budding (Figure 2). They are often seen in the cytoplasma of giant cells (*1*).

Diagnoses such as cryptococcosis and blastomycosis can be easily ruled out by direct examination or histopathology, but blastomycosis is unlikely in central and western African patients (*22*). The differential diagnosis is rarely difficult with cryptococcosis because of the shape and size of yeasts, presence of capsule, and lack of inflammation in the surrounding tissue. In any event, cryptococcal antigen testing and culture will easily ascertain the diagnosis.

Antigen detection in serum and urine is a sensitive test but has been developed for the variety *capsulatum*. It is validated in HIV-infected patients with disseminated diseases (*8*,*23*,*24*). *H. duboisii* is a cause of false-positive test results for antigen detection in urine. Antibody detection is useful for the retrospective diagnosis of histoplasmosis caused by variety *capsulatum*. Since variety *duboisii* antigens may cross-react with those of the variety *capsulatum*, serologic tests are potentially useful for diagnosis of African histoplamosis.

Although some PCR assays have been developed, they are not yet routinely used (*25*). Real-time and semi-nested PCR seem promising for the diagnosis of histoplasmosis due to variety *capsulatum* in blood and tissue samples (*26*–*28*). No PCR has yet been developed for variety *duboisii*, but a specific PCR assay could be helpful for this underdiagnosed disease.

Treatment of African histoplasmosis can be extrapolated from the guidelines of the Infectious Diseases Society of America established for histoplasmosis due to variety *caspulatum* (*29*). No clinical trial or efficacy studies have been performed for histoplasmosis due to variety *duboisii*, but as mortality rates are similar for the 2 species with the same management, the guidelines can be extrapolated to African histoplasmosis. In patients with AIDS, recommended therapy includes an intensive phase of 3 months with amphotericin B replaced by itraconazole (400 mg/d) for the severe forms, or itraconazole alone (600 mg/d for 3 days, then 400 mg/d) for mild forms. Fluconazole (800 mg/d) can be an alternative, but it has lower efficacy and a higher recurrence rate with isolates harboring higher MICs (*30*). Moreover, new azoles such as voriconazole require careful biologic and clinical monitoring when used for treating histoplasmosis in HIV-infected patients because of increased risk for in vitro resistance, especially in patients who had fluconazole (*31*). Nothing is known, however, about development of resistance for variety *duboisii*. Managing AIDS by HAART is an essential part of the treatment. The availability of HAART in Africa is increasing, but it may be absent in areas where histoplasmosis is endemic. This is a real concern for optimal management of such patients.

Maintenance therapy with itraconazole (200 mg or 400 mg/d) is recommended. Fluconazole (400 mg/d) should be avoided because of its reduced capacity to prevent relapses. However, as for many other opportunistic infections, maintenance therapy can be discontinued if the immunologic status of the patient improves, as described for case-patient 3. This patient’s prophylaxis was stopped 3 years ago, and she experienced no relapse and her CD4 count has always been >200/mm^3^. The stability of immune improvement has to be confirmed for several months before prophylaxis is stopped (*32*). Recent data suggest that the risk for relapse is rare after 12 months of treatment with a sustained immunologic improvement (CD4 >150/mm^3^) (*33*). However, in our experience based on the management of 20 cases of histoplasmosis due to variety *duboisii* in patients considered immunocompetent (*13*), relapses may be observed several years after the first episode. Thus, prolonged follow-up is mandatory for every patient with histoplasmosis due to variety *duboisii*.

Since HAART was introduced, the clinical and immunologic conditions of HIV-infected patients have dramatically improved, but physicians should now be aware of immune reconstitution inflammatory syndrome (IRIS) (*34*). As for many pathogens, both varieties of *H. capsulatum* can induce IRIS in HIV-infected patients, as recently reported by our group (*6*). The importance of the inflammatory reaction during IRIS contrasts with the mild one observed in the initial phase of the disease in severely immunocompromised patients and may require specific treatment.

Thus, histoplasmosis due to variety *duboisii* in HIV-infected patient remains a rare clinical entity but diagnosis should not be discounted because of the HIV status of the patient. Physicians working in Africa should be aware of *H. capsulatum* var. *duboisii* as a potentially emerging infection in HIV-infected patients.
